# Retrospective Longitudinal Survey on Canine Vector-Borne Pathogens: Trends and Challenges of 10 Years of Activities of a Veterinary Blood Bank

**DOI:** 10.3390/vetsci9060274

**Published:** 2022-06-06

**Authors:** Giulia Morganti, Arianna Miglio, Iolanda Moretta, Ambra L. Misia, Giulia Rigamonti, Valentina Cremonini, Maria T. Antognoni, Fabrizia Veronesi

**Affiliations:** Department of Veterinary Medicine, University of Perugia, Via San Costanzo 4, 06126 Perugia, Italy; giulia.morganti@unipg.it (G.M.); arianna.miglio@unipg.it (A.M.); iolanda.moretta@collaboratori.unipg.it (I.M.); ambralisa@hotmail.com (A.L.M.); giulia.rigamonti@studenti.unipg.it (G.R.); cremonini.valentina2@gmail.com (V.C.); fabrizia.veronesi@unipg.it (F.V.)

**Keywords:** canine vector-borne pathogens, veterinary blood bank, epidemiological data, 10 years activity

## Abstract

Canine vector-borne pathogens (CVBPs) represent a challenge for veterinary transfusion medicine, since some can be transmitted by blood transfusion and are of zoonotic concern. Epidemiological data on CVBPs, obtained during 10 years of pre-donor screening (2012–2021) by a veterinary blood bank in central Italy, were used to conduct a retrospective epidemiological longitudinal survey. The results were obtained using the Immunofluorescent Antibody Test (IFAT) conducted on sera in order to assess IgG antibodies against *Leishmania infantum*, *Ehrlichia canis*, *Anaplasma phagocythophilum*, *Babesia canis*, and *Rickettsia conorii*; the modified Knott’s test and an ELISA kit were used to detect *Dirofilaria immitis* and *Dirofilaria repens*. In total, 324 out of the 1260 canine blood donors (25.71%) tested seropositive for at least one pathogen. The highest overall positive rate was detected for *L. infantum* (12.22%), followed by *E. canis* (2.30%), *A. phagocytophilum* (1.19%), *D. repens* (0.95%), *D. immitis* (0.32%), and *B. canis* (0.16%). From 2012 to 2014, a prevalence of 20.12% was recorded for *R. conorii.* Mixed infections were recorded in 21 dogs. For all the CVBPs investigated, significant differences (*p* < 0.05) were not observed over the period studied. The results evidenced a non-negligible prevalence of CVBPs in canine donors, which were selected based on strict criteria concerning regular endo- and ectoparasite controls. The results confirmed that the blood bank could be a reliable local epidemiological observatory. The need for implemented screening is discussed.

## 1. Introduction

In the last few years, veterinary transfusion medicine has greatly expanded. It is well-known that transfusion carries the risk of adverse events, including transfusion-vector-borne transmitted infections (TTIs), from apparently healthy and asymptomatic blood donors [1,2,3].

Several guidelines have been developed to define the best protocols for detecting the most important microorganisms in veterinary medicine and to improve blood safety in different geographical areas [3,4,5,6,7,8,9,10]. However, the list of TTIs is not complete and needs continuous updating over the years based on the epidemiological spread of pathogens and also their impact on public health. Canine vector-borne pathogens (CVBPs) represent a challenge for veterinary transfusion medicine because many can potentially be transmitted by blood transfusion and are of zoonotic concern [11,12,13,14,15]. 

Canine vector-borne pathogens can be transmitted by a wide variety of blood-feeding arthropods such as fleas, ticks, mosquitoes, and sand flies, which have spread throughout Europe in the last decade [16]. The distribution pattern is affected by several biotic and abiotic factors, such as climate change, globalization, international trade, and the increasing contact between humans, dogs, and wildlife reservoir populations [15,17,18,19,20]. Furthermore, the large numbers of dogs traveling with owners, as well as the relocation of sheltered animals from endemic to previously non-endemic regions, have drastically contributed to the increasing number of canine vector-borne diseases (CVBDs) [21]. 

Most CVBDs are characterized by nonspecific, pleomorphic, and quite mild clinical signs; thus, animals frequently seem clinically healthy despite being infected and are potentially able to transmit pathogens if erroneously selected as blood donors. Veterinary blood banks could thus play a strategic role in the public health system in terms of monitoring and preventing CVBDs. 

Currently, the Italian guidelines (ItGL) for canine blood donors [22], drawn up by the Transfusion Study Group (GSTVet), recommend the investigation of CVBPs such as *Anaplasma phagocytophilum*, *Ehrlichia canis*, *Babesia* spp., transmitted by the bites of hard ticks of the Ixodidae family, as well as *Leishmania infantum* and *Dirofilaria* (i.e., *Dirofilaria immitis* and *Dirofilaria repens*), transmitted by sand-flies and mosquitos, respectively. 

The large number of canine donors recruited over several years of activity of veterinary blood banks could thus act as a real epidemiological observatory for these CVBPs in owned dogs living in specific areas. 

The aim of the present study was to conduct an epidemiological longitudinal survey on the major CVBPs of clinically healthy blood donor dogs in a veterinary blood bank of central Italy over a 10-year period (2012–2021).

## 2. Materials and Methods

### 2.1. Sampling Population

The database repository of the Emovet-Unipg blood bank of the Veterinary Teaching Hospital (OVUD) of the Department of Veterinary Medicine of Perugia (Umbria, central Italy) was searched for results concerning the pre-donor screening programme against CVBPs conducted over the decade 2012–2021. 

The canine blood donors included in the epidemiological survey came from central Italy, from areas where CVBPs are endemic [23,24,25]. The donors were selected using the suitability criteria indicated in the ItGL for body weight, age, general characteristics (e.g., regular vaccination with canine core and non-core vaccines, protection against endo- and ectoparasites), and physical examination. 

Informed consent was obtained from the owners of each donor before the ItGL mandatory screening programme, which involved clinicopathological examinations (e.g., complete blood count, typing of blood group, blood smears, serum chemistry, blood coagulation tests, urinalysis, and faecal examination) and investigations for CVBPs.

### 2.2. Pre-Donor Screening for CVBPs

The pre-donor screening for CVBPs involved serum detection and quantification of immunoglobulin G (IgG) against *L. infantum*, *E. canis*, *A. phagocythophilum*, *B. canis*, and *R. conorii* (only from February 2012 to December 2014) using an indirect fluorescent antibody test (IFAT) [19], and antigen and microfilariae detection of *Diroflaria* spp., according with the Office International des Epizooties (OIE) manual of standards for diagnostic tests and vaccine.

The presence of IgG against *R. conorii*, *E. canis*, *B. canis*, and *A. phagocytophilum* was assessed by IFAT using commercial antigens, i.e., slides coated with purified individual substrate antigens (MegaFluo^®^ *Rickettsia conorii*, MegaFluo^®^ *Ehrlichia canis*, MegaFluo^®^ *Babesia canis*, MegaFluo^®^ *Anaplasma phagocytophilum*, MegaCor Diagnostik GmbH, Horbranz, Austria). For the detection of anti-*Leishmania* IgG, sera were tested with a homemade IFAT following the standard procedures recommended by the Office International des Epizooties [26,27] and using promastigotes of *L. infantum* zymodeme MON-1 (MHOM/TN/80/IPT-1) as antigenic source.

For all the serological tests, commercial anti-canine IgG polyclonal antiserum conjugated to fluorescein isothiocyanate (MegaFluo^®^ FITC IgG, MegaCor Diagnostik GmbH, Horbranz, Austria; working dilution 1/100) was used as a conjugate. Positive and negative controls provided by the commercial kits were added to each specific reaction for *R. conorii*, *B. canis*, *E. canis*, and *A. phagocytophilum*. However, positive and negative controls for *Leishmania* were used consisting of sera obtained from a cytologically-confirmed clinically ill dog, and from a dog that had previously tested negative on serological and molecular assays, respectively. 

The results obtained were interpreted using the cut-off dilutions of 1/25 for *E. canis*, 1/64 for *B. canis*, 1/80 for *L. infantum*, *A. phagocytophilum* and *R. conorii*. Two-fold serial dilutions were prepared and tested to define the serum titre of samples testing positive at screening. 

Serum circulating antigens for *D. immitis* were determined with the Dirochek^®^ Heartworm Antigen Test (Zoetis Inc., Kalamazoo, MI, USA) according to the manufacturer’s instructions. One millilitre of blood in ethylenediaminetetraacetic acid (EDTA) was analysed with the modified Knott test and Giemsa staining for microfilariae detection. The identification of microfilariae was based on their morphology and morphometry using the key reported by Euzeby [28].

### 2.3. Statistical Analysis 

The prevalence referring to each CVBP was computed with the associated 95% confidence interval (95% CI) both for the overall period and for each single year. Inferential analysis was performed to compare CVBPs positive rates over the decade (*p* < 0.05). Statistical analyses were performed using commercial software (SPSS, Version 22.0; Chicago, IL, USA).

### 2.4. Ethical Statement

Informed consent was obtained from the owners of dog candidate blood donors, as required by the Blood Bank to become eligible donors. The program for donor screening included the collection of information regarding the medical history of the dogs and infectious disease testing as suggested by the guidelines from the Italian Ministry of Health.

## 3. Results

Pre-donor screening data of 1260 dogs were included in the retrospective longitudinal CVBPs survey. An annual average of 126 dogs (s.d. 52.73) were enrolled, with the highest number in 2014 (i.e., 210), while in 2020 and 2021 the numbers were quite low (77 and 55, respectively) because of COVID-19 restrictions. 

A total of 324 (25.71%, 95% CI: 23.30–28.13%) of the 1260 dogs were found to be seropositive for at least one pathogen. Table 1 shows the CVBPs prevalence and respective antibody titers. The highest overall positive rate was detected for *L. infantum* (154 dogs, 12.22%, 95% CI: 10.41–14.03%), followed by *E. canis* (29 dogs, 2.30%, 95% CI: 1.47–3.13%), *A. phagocytophilum* (15 dogs, 1.19%, 95% CI: 0.59–1.79%), *D. repens* (12 dogs, 0.95%, 95% CI: 0.42–1.49%), *D. immitis* (4 dogs, 0.32%, 95% CI 0.01–0.63%), and *B. canis* (2 dogs, 0.16%, 95% CI: 0.00–0.38%). 

From 2012 to 2014, a prevalence of 20.12% (95% CI: 16.72–23.50%) was recorded for *R. conorii* (108 dogs). Mixed infections were recorded in 21 dogs (i.e., 13 *L. infantum* and *R. conorii*, 2 *L. infantum* and *E. canis*, 1 *L. infantum* and *A. phagocytophilum*, 1 *L. infantum* and *B. canis*, 1 *L. infantum*, *E. canis* and *R. conorii*, and 3 *E. canis* and *R. conorii*). 

Knott’s test revealed *D. repens* and *D. immitis* microfilariae in 12 and 2 dogs, respectively; however, no mixed infections were recorded, and neither were other haematic microfilariemic filarial nematodes (e.g., *Acanthocheilonema reconditum*) recovered.

In the longitudinal survey, *L. infantum* seropositivity rates ranged from 3.6% to 17.3% with an average prevalence of 10.5% (s.d. 4.87). These results varied consistently over the sampling years, with the highest rates in the first 6 years and a progressive decrease in the last 4 years. *E. canis* ranged from 0% to 8%, with an average prevalence of 2.16% (s.d. 2.60); low seropositivity was recorded for *A. phagocytophilum* (0–3.6%), with an average prevalence of 0.97% (s.d. 1.57). The positivity rates of *B. canis* range between 0% to 1%, with an average prevalence of 0.16% (s.d. 0.35). Positive values for *D. immitis* were overall low, ranging from 0% to 0.9%, while *D. repens* ranged from 0.92% to 1.02%. 

The trend of CVBPs rates over the decade of the blood bank’s activities is reported in Table 2.

For all the investigated CVBPs, statistically significant differences (*p* < 0.05) were not observed across the study period. Considering the data collected over 3 years, no prevalence trend was evaluated for *R. conorii*. 

## 4. Discussion

This retrospective longitudinal survey provides further knowledge regarding important CVBPs in central Italy, exploiting the data obtained from pre-donor screenings collected in the repository of a veterinary blood bank.

Although the potential blood donors were selected according to suitability criteria that include regular ectoparasite controls with anti-feeding/insecticidal products against the main vectors, and despite the introduction and commercialization in the last decade of a wide range of products containing pyrethroids and isoxazolines that improve the control of ectoparasites, a non-negligible seroprevalence of CVBPs was found in the blood donors.

An overall seroprevalence of 12.22% was detected for *L. infantum*, which is slightly higher than that found in a recent study [29]. From 2012–2021, the positivity rates, which ranged from 3.6% to 17.3%, were highest in the first 6 years and then decreased, although not significantly (*p* > 0.05). This trend does not reflect the real seroprevalence of Canine Leishmaniosis (CanL) in the investigated areas. In fact, *L. infantum* is endemic in the central region investigated here [23], showing various levels of endemicity (from low to medium) depending on the geographical area. In addition, the average prevalence of infection has increased in the last decade from seroprevalence rates of less than 10% [30] to over 15% [23]. It is thus conceivable that the selection criteria used for candidate blood donors may have affected our results and may have contributed to the selection of a “lower risk” population (e.g., exclusion of owners with several dogs, of which some have tested positive for CanL).

In the present study, 95 out of the 154 *L. infantum* seropositive dogs presented a questionable titer of 1/80. This finding highlights how in endemic areas it might be difficult to select blood donors, since many animals may show low antibody titers as the expression of seasonal exposition, and thus should be temporarily excluded. In fact, to date, the ItGL recommendation in the case of seroreactivity for *L. infantum* consists in waiting and reconsidering the dog after a month or two and after a negative result. 

Further diagnostic strategies, including molecular techniques on sensitive targets (e.g., conjunctival swabs), should be discussed and recommended in the near future by the ItGL in order to select blood donors living in or coming from CanL endemic areas. This would help to differentiate between infected dogs in a preclinical phase of CanL and those that only had contact with the parasite.

Although the climate and environmental characteristics of the investigated areas, as well as the strong hunting culture, can greatly influence the presence and spread of tick-borne pathogens (TBPs) [31], the overall seroprevalence detected here was moderate. 

Canine monocytic ehrlichiosis (CME) due to *E. canis* has a significant clinical impact on dogs [32] and is traditionally considered as being the most widespread in central Italy [31,33]. The results obtained in the present work evidenced a moderate circulation of *E. canis* (2.3%) during the longitudinal study period. These data are in line with a recent investigation with dogs subjected to different preventative regimens for ectoparasites in central and southern Italy, in which a 2.1% prevalence was detected for *E. canis* [34], which is slightly lower than that reported in a recent study on suitable blood donors [29]. 

*Anaplasma phagocytophilum*, the causative agent of human and canine granulocytic anaplasmosis (CGA), was detected in 1.19% of the analysed dog sera. This overall prevalence rate is in line with previous seroepidemiological surveys conducted in the same areas, which showed prevalence rates from 2% to 4.6% [29,31,34,35] and confirmed exposure to *Ixodes ricinus* bites in our canine population. It is possible that the serological positivity in this selected category could also be due to the major activity of the vector *I. ricinus* during milder winters/warm springs, which are periods of the year when the use of antiparasitic drugs is overlooked by dog owners. 

During the study period, a prevalence rate of 0.16% was found for *B. canis*, evidencing a rather low pathogen circulation in the areas studied compared with the 4% serological positivity reported by other authors [34,36].

*Rickettsia conorii* seropositivity was assessed only within the first 3 years of the study period, showing higher positivity rates compared with the other tested CVBPs. According to ItGL, testing is not mandatory, and so the serological screening was interrupted. In the study by Vascellari et al. [37] on the exposure to VBPs in candidate blood donors and free-roaming dogs in northeast Italy, the most frequent pathogens belonged to members of the genus *Rickettsia*. Similar results were recorded in a recent study by Colombo et al. [34], who highlighted that *R. conorii* was still the most widely distributed *Rickettsia* species in dogs throughout the Mediterranean basin. Recent studies have also confirmed the non-negligible exposure of the human and canine population to spotted fever group rickettsioses in the areas of central Italy investigated here [25,38]. 

Although not mandatory according to ItGL, serology for *R. conorii* should be encouraged, since dogs represent sensitive sentinels in assessing the infective pressure of this zoonotic pathogen. Serology should thus be reconsidered in the near future by the GSTVet, given the dual role of veterinary blood banks: ensuring the safety of donor and recipient patients [29], and indirectly safeguarding public health.

Despite the introduction and consolidation of new competent vectors (i.e., *Aedes albopictus*) in the last decade, the extensive use of macrocyclic lactones (MLs) has significantly reduced the prevalence of *D. immitis* and *D. repens* infestations in the investigated areas, in which, however, they remain endemic. An extended epidemiological survey of owned dogs conducted at the national level showed a prevalence rate for *D. immitis* in Umbria of 2% and higher for *D. repens* (6%), with mainly asymptomatic and pauciparasitic infestations and co-infestations in about 75% of cases [39].

In the present study, lower positivity rates (0.95% for *D. repens*, 0.32% for *D. immitis*) were detected. Although few dogs were submitted to specific chemoprophylaxis for cardiopulmonary and or/subcutaneous dirofilariosis, the wide use of MLs for internal parasite control, included in the suitability criteria to become blood donors, could have influenced the low prevalence rates. 

We recommend using the modified Knott test to reveal specific microfilaremia, since the ELISA test used to reveal positivity towards *D. immitis* is inadequate for *D. repens*; therefore, this infestation could be underestimated with significant public health implications. 

In fact, in Europe, and especially in the Mediterranean Basin, *D. repens* is the main agent of human dirofilariosis, especially in ocular forms [29,40,41,42,43].

## 5. Conclusions

The epidemiological data acquired through the retrospective consultations used in the present work highlight the role that canine blood donors and blood banks could play as a reliable local epidemiological observatory for major CVBPs, especially for those of zoonotic concern in terms of assessing the risk of human exposition.

The prevalence rates detected confirm the need to continue and improve the standard donor selection criteria and to implement the screening protocols recommended by the experts of the GSTVet through the use of sensitive tests, also considering the possible implications for transfusion veterinary medicine and public health.

## Figures and Tables

**Table 1:** Parasitological results of CVBPs detected in blood donor dogs during a decade of blood bank activities (2012–2021).

			End Point Titres (IgG)			
CVBPs	Diagnostic Methods	Number of Dogs Positive; % (95% CI)	1/80 (n Dogs)	1/160 (n Dogs)	1/320 (n Dogs)	1/640 (n Dogs)
***Leishmania infantum***	IFAT	154 12.22% (10.41–14.03%)	95	25	23	11
***Rickettsia conorii***	IFAT	108 20.15% * (16.72–23.50%)	102	5	1	0
***Anaplasma phagocytophilum***	IFAT	15 1.19% (0.59–1.79%)	7	1	4	2
			**1/25** **(n Dogs)**	**1/50** **(n Dogs)**	**1/100** **(n Dogs)**	**1/200** **(n Dogs)**
***Ehrlichia canis***	IFAT	29 2.30% (1.47–3.13%)	17	5	5	2
			**1/64** **(n Dogs)**	**1/128** **(n Dogs)**	**1/256** **(n Dogs)**	**1/512** **(n Dogs)**
***Babesia canis***	IFAT	2 0.16% (0.00–0.38%)	2	0	0	0
***Dirofilaria repens***	Knott test	12 0.95% (0.42–1.49%)	-	-	-	-
***Dirofilaria immitis***	Knott test/ELISA	4 0.32% (0.01–0.63%)	-	-	-	-

* Data from 2012 to 2014; -: Not expected by the method used; CI: Confidence interval; IFAT: indirect fluorescent antibody test.

**Table 2:** Trend of CVBPs rates over a decade of blood bank activities (2012–2021).

	Year 2012	Year 2013	Year 2014	Year 2015	Year 2016	Year 2017	Year 2018	Year 2019	Year 2020	Year 2021
***N. of dogs***	118	208	211	152	143	119	91	88	77	55
***Leishmania infantum*** **n, %** **(95% CI)**	14 11.86% (6.03–17.70%)	36 17.30% (12.17–22.45%)	32 15.17% (10.33–20.01%)	23 15.13% (9.43–20.83%)	19 13.28% (7.72–18.85%)	12 10.08% (4.67–15.49%)	8 8.79% (2.97–14.61%)	4 4.55% (0.19–8.90%)	4 5.20% (0.24–10.15%)	2 3.64% (0.00–8.58%)
***Ehrlichia canis*** **n, %** **(95% CI)**	4 3.38% (0.12–6.66%)	3 1.44% (0.00–3.06%)	5 2.38% (0.32–4.42%)	7 4.60% (1,27–7.94%)	0 0.00% (0.00–0.00%)	9 8.00% (2.81–13.19%)	0 0.00% (0.00–0.00%)	0 0.00% (0.00–0.00%)	0 0.00% (0.00–0.00%)	1 1.82% (0.00–5.35%)
***Anaplasma phagocytophilum*** **n, %** **(95% CI)**	0 0.00% (0.00–0.00%)	0 0.00% (0.00–0.00%)	0 0.00% (0.00–0.00%)	0 0.00% (0.00–0.00%)	4 2.80% (0.09–5.50%)	4 3.60% (0.60–6.60%)	1 1.00% (0.00–3.24%)	3 3.44% (0.00–7.20%)	2 2.60% (0.00–6.15%)	1 1.82% (0.00–5.35%)
***Babesia canis*** **n, %** **(95% CI)**	0 0.00% (0.00–0.00%)	0 0.00% (0.00–0.00%)	0 0.00% (0.00–0.00%)	1 0.60% (0.00–1.94%)	0 0.00% (0.00–0.00%)	0 0.00% (0.00–0.00%)	1 1.00% (0.00–0.00%)	0 0.00% (0.00–0.00%)	0 0.00% (0.00–0.00%)	0 0.00% (0.00–0.00%)
***Dirofilaria immitis*** **n,%** **(95% CI)**	1 0.84% (0.00–2.50%)	1 0.48% (0.00–1.42%)	0 0.00% (0.00–0.00%)	0 0.00% (0.00–0.00%)	1 0.70% (0.00–2.07%)	1 0.90% (0.00–2.48%)	0 0.00% (0.00–0.00%)	0 0,00% (0.00–0.00%)	0 0.00% (0.00–0.00%)	0 0.00% (0.00–0.00%)
***Dirofilaria repens*** **n,%** **(95% CI)**	0 0.00% (0.00–0.00%)	2 1.00% (0.00–2.29%)	4 1.90% (0.06–3.74%)	0 0.00% (0.00–0.00%)	0 0.00% (0.00–0.00%)	2 1.70% (0.00–3.99%)	2 2.20% (0.00–5.21%)	1 1,14% (0.00–3.35%)	1 1.30% (0.00–3.83%)	0 0.00% (0.00–0.00%)
***Rickettsia conorii*** **n,%** **(95% CI)**	24 20.33% (13.08–27.60%)	47 22.60% (16.91–38.38%)	37 17.54% (12.40–22.67%)	n.p.	n.p.	n.p.	n.p.	n.p.	n.p.	n.p.

n.p.: Not performed; CI: Confidence interval.

## Data Availability

All study data are presented in the article.
